# Comparative Analysis of Gut Microbiota in Eri Silkworm (*Samia ricini*) Larvae Fed on Different Food Plants

**DOI:** 10.3390/insects17060553

**Published:** 2026-05-27

**Authors:** Yu Guo, Xiangbiao Liu, Yalei Wang, Huiduo Guo, Heying Qian

**Affiliations:** 1 Jiangsu Key Laboratory of Sericultural and Animal Biotechnology, School of Biotechnology, Jiangsu University of Science and Technology, Zhenjiang 212100, China; 2 Key Laboratory of Silkworm and Mulberry Genetic Improvement, Ministry of Agriculture and Rural Affairs, Sericultural Scientific Research Center, Chinese Academy of Agricultural Sciences, Zhenjiang 212100, China

**Keywords:** *Samia ricini*, diets, gut microbiota, digestive enzyme activity, growth performance

## Abstract

Diet plays a critical role in shaping the composition of gut microbiota in insects. *Samia ricini*, an economically important Lepidoptera insect, is a polyphagous herbivore that offers a useful model for studying dietary effects on the animal gut microbiome. Here, we fed *S. ricini* larvae with different food plants, *Ricinus communis*, *Ailanthus altissima*, and *Manihot esculenta* leaves to investigate how host plant species influence growth performance, digestive enzyme activities, and the gut microbial community. Our results showed that the *Ricinus* group exhibited better growth performance. Regarding digestive enzymes, the midgut lipase activity was significantly higher in the *Ricinus* group than in the *Ailanthus* group, while no significant differences were observed in α-amylase, cellulase, or trypsin activities among the three groups. Compared to the *Manihot* group, the *Ricinus* group showed increased bacterial richness, while the *Ailanthus* group showed increased bacterial diversity. β-diversity analysis further revealed distinct microbial community structures among all three dietary groups. Specifically, *Acinetobacter*, *Mammaliicoccus*, *Roseateles*, *Methylobacterium*, *Agrobacterium*, *Faecalibacterium*, and *Segatella* were the dominant bacterial genera. Functional prediction revealed that gut microbes enriched in the *Ricinus* group were associated with terpenoid/polyketide metabolism, xenobiotics biodegradation, and glycan biosynthesis, whereas those involved in carbohydrate metabolism and biosynthesis of other secondary metabolites were higher in the *Manihot* group. Spearman correlation analysis indicated that *Methylobacterium*, *Methylorubrum*, and *Agrobacterium* were significantly positively correlated with larval weight, while *Staphylococcus* and *Cyanothece_PCC-7424* exhibited negative correlations. Collectively, these findings suggest a potential association between different plant-derived diets, gut microbiota composition, and host growth performance, highlighting the pivotal role of diet in shaping insect gut microbial communities.

## Simple Summary

Gut microbiota play an important role in assisting insects in quickly adapting to host plants. *Samia ricini* is an economically important polyphagous lepidopteran insect. However, the relationship between gut microflora and larval growth performance in response to short-term diet switching in *S. ricini* remains unclear. Larvae reared on tree-of-heaven leaves showed poorer growth and lower midgut lipase activity compared to those fed on castor leaves. Short-term diet switching significantly altered gut bacterial richness, diversity, and community structure, and reshaped microbial functional profiles in *S. ricini*. *Methylobacterium* was closely correlated with larval weight. This study reveals that short-term diet switching affects larval growth, associated with the alterations in gut microbiota composition and function in *S. ricini*.

## 1. Introduction

Insect guts harbor numerous microorganisms, including bacteria, fungi, archaea, and viruses [1,2], which primarily colonize the host’s digestive tract and play fundamental roles in sustaining host physiology [3,4,5]. Gut microbiota contribute essential digestive enzymes for breaking down complex carbohydrates in the diet, thereby supporting insect growth and development [5]. Beyond nutritional contributions, they further modulate critical host processes such as host behavior [6], detoxification [7], and immune regulation [8,9]. Indeed, insects rely profoundly on their gut microbiota for survival and the maintenance of normal physiological functions [10].

The composition and abundance of gut microbiota are influenced by multiple factors, such as host species, environmental conditions, developmental stage, and immune response [6]. Among these, diet acts as a principal driver in the formation of intestinal microbial communities. Microbiota plasticity allows insects to adapt to diverse diets. This is particularly evident during larval stages, where the gut bacterial community exhibits high sensitivity to dietary and environmental fluctuations. Previous studies indicate that dietary variations directly reshape the structure and composition of gut microbiota, which in turn regulate key host functions such as nutrition acquisition, metabolic homeostasis, and immune defense [11,12].

In Saturniidae, *Samia ricini* represents the only fully domesticated species of significant economic importance, and is now reared across several countries. Unlike the lustrous silk produced by *Bombyx mori*, eri silk possesses a wool-like and matte texture. Beyond its traditional textile value, the fibroin derived from eri silk has garnered increasing interest in biomedical fields. Its inherent biocompatibility enables applications in advanced wound dressings, controlled drug delivery systems, and tissue engineering scaffolds [13,14,15,16]. *S. ricini* is a polyphagous insect, feeding on more than 30 plant species from distantly related taxonomic families [17]. Among these, it primarily feeds on *Ricinus communis* leaves, while *Manihot esculenta* leaves act as its secondary food source. Additionally, it has been reported that *Ailanthus altissima* leaves act as a plant capable of hosting larvae of *S. ricini* [17]. Because of its higher survival rate and greater adaptability to diverse host plants under artificial rearing conditions compared to the silkworm *B. mori*, *S. ricini* has received increasing attention as another potential novel model of Lepidoptera. Diet was found to exert a dominant influence on relative larval growth rate, survivability, and cocoon parameters of *S. ricini* [17]. Gut bacteria are known to assist insects in quickly adapting to host plants. However, most previous studies, including those on *S. ricini*, have focused on long-term host plant adaptation or comparisons across different developmental stages. However, the effects of short-term diet switching on the composition of gut microflora and larval growth performance in *S. ricini* remain unclear.

The study investigated the relationship between gut microbiota composition and larval performance in response to short-term host plant switching in *S. ricini.* We compared the larval growth performance and digestive enzyme activities across different dietary shift treatments, and analyzed the correlations between microbial community composition and host growth performance. This study deepens our understanding of gut bacteria-mediated insect–plant interactions.

## 2. Materials and Methods

### 2.1. Experimental Materials

*S. ricini* were obtained from the National Genetic Resources Bank of Silkworm in Zhenjiang, China. All larvae were reared in a rearing room at the Research Station of the Sericulture Institute, Jiangsu University of Science and Technology, at 27 ± 2 °C and 80 ± 10% relative humidity. Larvae were reared in disposable plastic crispers (30 cm × 25 cm × 10 cm) with perforated lids for ventilation at a density of 30 larvae per container. From the first instar to the end of the fourth instar, all larvae were fed exclusively with fresh *Ricinus communis* leaves. At the beginning of the fifth instar, the larvae were randomly assigned to three experimental groups (each with three biological replicates of 30 larvae), which were fed with leaves of *Ricinus communis*, *Ailanthus altissima*, or *Manihot esculenta*, designated as the *Ricinus* group, *Ailanthus* group, and *Manihot* group, respectively. See Table 1. All three types of leaves were collected from the experimental station of the Sericulture Institute, Jiangsu University of Science and Technology. Before feeding, all leaves were washed with tap water and air-dried. During the fifth instar, larvae were provided with fresh leaves twice daily; before each feeding, remaining leaves and frass were removed to maintain a clean and dry environment. The rearing conditions followed standard protocols for *S. ricini* used in our institute [18].

### 2.2. Sample Collection and Processing

Food intake and body weight of larvae in each group were recorded from day 1 to day 6 of the 5th instar. Based on preliminary observations, day 3 of the 5th instar was selected for sampling because it is the stage of peak food intake and growth, and significant differences in body weight among groups had already emerged by this time. On day 3 of the 5th instar, six *S. ricini* were randomly selected from each of the *Ricinus*, *Ailanthus*, and *Manihot* groups. To minimize interference from food residues, all fifth-instar larvae were subjected to a 24 h starvation period before dissection [19]. After starvation, the larvae were placed on ice for approximately 5–10 min until completely immobilized and rigid, then washed with 70% alcohol to minimize external microbial contamination. The midgut contents were then collected directly into 2 mL sterile Eppendorf tubes. All samples were stored at −80 °C until use.

### 2.3. Determination of Digestive Enzyme Activity

The activities of α-amylase (AMS), lipase (LPS), trypsin (Try), and cellulase (CL) of the midgut were determined by commercial kits according to manufacturers’ instructions. The AMS activity was measured using an α-Amylase Assay Kit (Nanjing Jiancheng, Nanjing, China) at 660 nm. The LPS activity was measured using a Lipase Assay Kit (Nanjing Jiancheng) at 580 nm. The Try activity was measured using a Trypsin Assay Kit (Suzhou Comin, Suzhou, China) at 555 nm. The CL activity was measured using a Cellulase (CMCase) Activity Assay Kit (Suzhou Comin) at 620 nm.

### 2.4. DNA Extraction and 16S rDNA Sequencing

The midgut content bacterial DNA was extracted using the QIAamp FAST DNA Stool Mini Kit (QIAGEN, Hilden, Germany) as described previously [20]. The universal primer set 341F (5′-CCTACGGGNGGCWGCAG-3′) and 806R (5′-GGACTACHVGGGTATCTAAT-3′) was used to amplify the V3-V4 hypervariable region of bacterial 16S rDNA. And the PCR amplification was verified by agarose gel extraction. PCR products with the correct size were purified using the AMPure XP Beads (Beckman Coulter, Brea, CA, USA) according to the manufacturer’s instructions and then pooled for sequencing library preparation. The Illumina NovaSeq 6000 platform (Illumina, San Diego, CA, USA) was used for 16S rDNA sequencing.

### 2.5. Bioinformatic Analysis

The raw reads were deposited into the NCBI Sequence Read Archive (SRA) database. The DADA2 software (version 1.14.1) was used for data filtering to obtain high-quality clean raw data [21]. Taxonomic units with a relative abundance of ≥1% from the phylum to genus level, as well as OTUs were defined as the dominant group. These dominant groups were screened out and ranked by their average abundance for subsequent statistical analysis. Alpha diversity indices, including species richness (Chao1) and community diversity (Shannon and Simpson), were calculated using the free online Omicshare Cloud Platform. Principal coordinate analysis (PCoA) based on the Jaccard distance metric and the Adonis test were also conducted on the same platform. To identify microbial biomarkers with significant differences and high discriminatory power among groups, we performed LEfSe analysis with an LDA score threshold set to 4.0.

### 2.6. Statistical Analysis

Statistical analyses for enzyme activity levels and growth indicators were carried out by using SPSS 19.0 software (IBM SPSS, Chicago, IL, USA). Data were presented as means ± SEM. *p*-values were calculated using one-way analysis of variance (ANOVA) with Tukey’s post hoc test between groups. Microbiome data are typically non-normally distributed, and the conventional practice is to use Spearman’s rank correlation coefficient. Correlation analyses among microbial taxa, digestive enzyme activities, and growth indicators were performed using Spearman correlation analysis. *p* < 0.05 was considered statistically significant.

## 3. Results

### 3.1. S. ricini Fed on R. communis Leaves Exhibited Better Growth Performance than Those Fed on Ailanthus and Manihot Leaves

**Figure 1:**
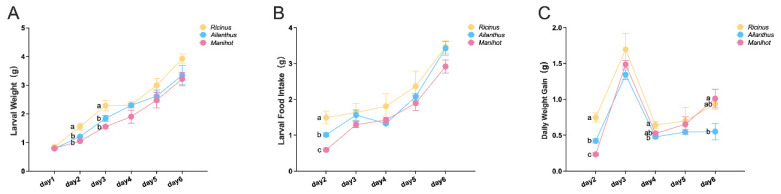
Growth and development indicators of *Samia ricini* fed on different food plants: *Ricinus communis* leaves (*Ricinus* group); *Ailanthus altissima* leaves (*Ailanthus* group); *Manihot esculenta* leaves (*Manihot* group). (**A**) Larval weight; (**B**) Food intake; (**C**) Daily weight gain. The data are presented as the mean ± SEM. Different letters above bars indicate statistically significant differences among the groups (*p* < 0.05).

To investigate the effects of *R. communis* leaves, *A. altissima* leaves, and *M. esculenta* leaves on the growth of *S. ricini*, we recorded the larval weight, feed intake, and daily weight gains during the 5th instar. The results showed that the *Ricinus* group at day 2 and day 3 of the 5th instar (L5D2 and L5D3) exhibited significantly higher larval weight, accompanied by increased food intake and daily weight gains at day 2 among the three groups (Figure 1A–C). After adaptation to the diets for three days, the *Ailanthus* and *Manihot* groups showed comparable food intake to the *Ricinus* group (Figure 1B). No significant difference in body weight was found among the groups during the late 5th instar (day 4–day 6). However, the *Ricinus* group exhibited the highest body weight with a significantly greater daily weight gain at day 4 (Figure 1A,C). Collectively, the results indicated *S. ricini* fed on *R. communis* leaves exhibited better growth performance than those fed on *Ailanthus* or *Manihot* leaves.

### 3.2. Midgut Digestive Enzyme Activities of S. ricini Larvae

**Figure 2:**
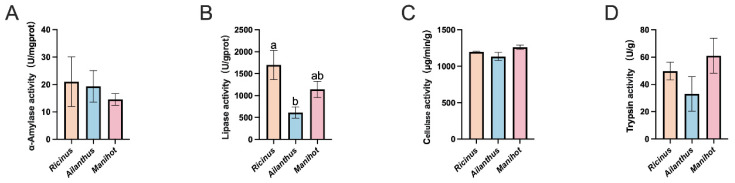
Digestive enzyme activity in the midgut of *Samia ricini*. (**A**) α-Amylase activity; (**B**) Lipase activity; (**C**) Cellulase activity; (**D**) Trypsin activity. The data are presented as the mean ± SEM. Different letters above bars indicate statistically significant differences among the *Ricinus*, *Ailanthus*, and *Manihot* groups (*p* < 0.05).

Next, the activities of digestive enzymes, including α-amylase, lipase, cellulase, and trypsin, in the midgut on day 3 was measured using ELISA kits. There were no significant differences in the activity of α-amylase, cellulase, and trypsin among the three groups (Figure 2A,C,D). Lipase activity was significantly higher in the *Ricinus* group than in the *Ailanthus* group, while the *Manihot* groups showed no significant difference compared to either the *Ricinus* or *Ailanthus* groups (Figure 2B). The results showed that *S. ricini* larvae feeding on *R. communis* leaves showed a higher lipase activity.

### 3.3. Effects of Different Plant Diets on Midgut Microbiota Abundance and Diversity in S. ricini

**Figure 3:**
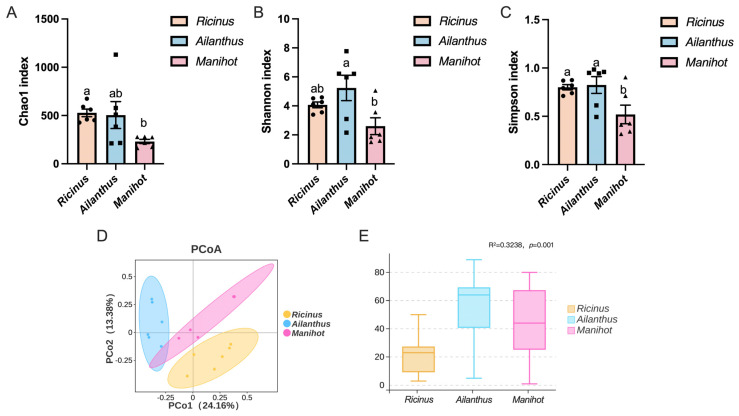
Effects of three food plant leaves on the bacterial diversity in *S. ricini*. (**A**–**C**) The α-diversity of the midgut microbiota in the three groups was evaluated using the Chao1, Shannon and Simpson indices. (**D**) PCoA analysis based on jaccard distance matrix. (**E**) Statistical analysis of Adonis test among three groups. Different letters above bars indicate statistically significant differences among the *Ricinus*, *Ailanthus*, and *Manihot* groups (*p* < 0.05).

To explore the effects of different food plants on the microbiota richness and diversity in the midgut of *S. ricini* larvae, 16S rDNA gene sequencing was applied. The Chao1 index in the *Manihot* group was significantly lower than that in the *Ricinus* group. However, there was no significant difference between the *Ailanthus* and *Manihot* groups (Figure 3A). The Shannon index showed that the *Ailanthus* group was significantly higher than the *Manihot* group, while the *Ricinus* group showed no significant difference from either the *Ailanthus* or *Manihot* groups (Figure 3B). Simpson index showed that both the *Ricinus* and *Ailanthus* groups were significantly higher than the *Manihot* group, with no significant difference between the *Ricinus* and *Ailanthus* groups (Figure 3C). Principal coordinate analysis (PCoA) based on the Jaccard distance metric showed that the bacterial communities were separated from each other among the three groups (Figure 3D). Furthermore, the Adonis analysis was performed to evaluate whether group differences were significantly greater than within-group differences, with results indicating significant differences in community structure among the three groups (R^2^ = 0.3238, *p* = 0.001) (Figure 3E). The PCoA and Adonis analyses together showed that there was significant heterogeneity in the microbial community structure among the *Ricinus*, *Ailanthus*, and *Manihot* groups. Overall, both the *Ricinus* and *Ailanthus* groups supported higher levels of either richness or diversity compared to the *Manihot* group.

### 3.4. Midgut Microbial Composition and Relative Abundance in S. ricini Fed on Different Food Plants

**Figure 4:**
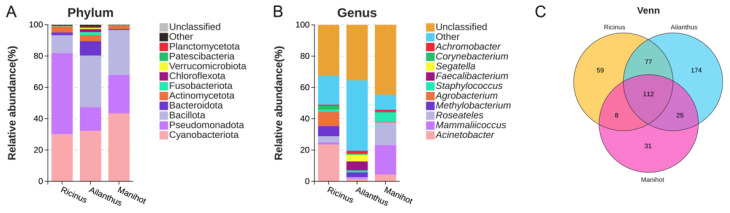
Composition and relative abundance of midgut microbiota in *S. ricini* fed three plant-based diets at different taxonomic levels. (**A**,**B**) Phylum and Genus levels of relative abundance; (**C**) Genus’s level OTU Venn diagram.

The composition and relative abundance of midgut microbiota of the three groups at phylum and genus taxonomic levels are shown in Figure 4. At the phylum level, a total of 14 bacterial phyla were identified from the three groups, with Cyanobacteriota, Pseudomonadota, Bacillota, and Bacteroidota as the dominant phyla and *S. ricini* fed on *R. communis* leaves had the highest relative abundance of Pseudomonadota, while the *Ailanthus* group had the highest relative abundance of Bacteroidota (Figure 4A). At the genus level, 94 bacterial genera were identified across the three groups. The dominant genera included *Acinetobacter*, *Mammaliicoccus*, *Roseateles*, *Methylobacterium*, *Agrobacterium*, *Faecalibacterium*, and *Segatella*. Distinct enrichment patterns were observed among the three groups. The *Ricinus* group showed enrichment of *Acinetobacter*, *Roseateles*, *Methylobacterium*, and *Agrobacterium*. The *Ailanthus* group was enriched for *Methylobacterium*, *Faecalibacterium*, and *Segatella*. In contrast, the *Manihot* group was characterized by *Mammaliicoccus*, *Roseateles*, *Staphylococcus*, and *Acinetobacter*. These group-specific enrichments reveal significant differences in bacterial community composition (Figure 4B). In addition, Venn diagram analysis identified a total of 112 common genera of the three groups, and the *Ricinus* group contained 59 unique genera, while the *Ailanthus* and *Manihot* groups had 174 and 31 unique genera, respectively (Figure 4C). Collectively, these results revealed profound diet-induced alterations in the midgut microbiota.

### 3.5. Potential Midgut Microbiota Biomarkers Defined by LEfSe

**Figure 5:**
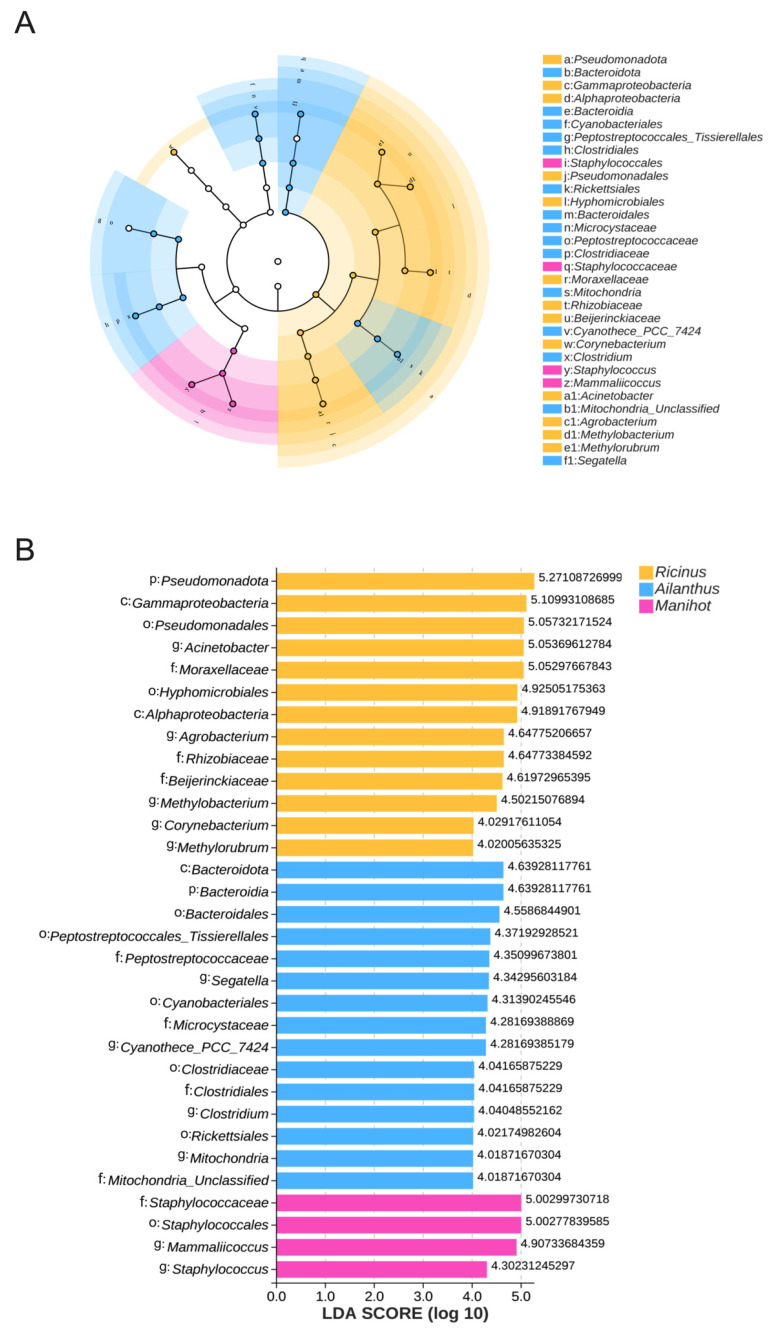
Potential microbiota biomarkers were identified using LEfSe in *S. ricini* larvae fed on different food plants. Potential midgut microbiota biomarkers as defined by LEfSe. Differentially abundant features are shown by the Cladogram (**A**) and LDA scores (**B**) among the three groups.

Using a threshold LDA score of 4.0 for feature recognition, the relative abundance of different midgut microbiota taxa, from phylum to genus, in the three groups was evaluated using linear discriminant analysis effect size (LEfSe). As shown in Figure 5, a total of 32 biomarkers were identified across the three groups. The *Ricinus* group harbored 13 significantly enriched bacterial taxa, including the biomarker genera *Acinetobacter*, *Agrobacterium*, *Methylobacterium*, *Corynebacterium*, and *Methylorubrum*. In contrast, the *Ailanthus* group exhibited 15 enriched taxa. Among these, *Segatella*, *Cyanothece_PCC-7424*, and *Clostridium* were significantly enriched at the genus level. The *Manihot* group contained 4 biomarkers, in which *Mammaliicoccus* and *Staphylococcus* are significantly enriched. These microbiota, undergoing drastic diet-induced changes, may in turn affect the metabolic function of the midgut.

### 3.6. Functional Prediction of Midgut Microbiota

**Figure 6:**
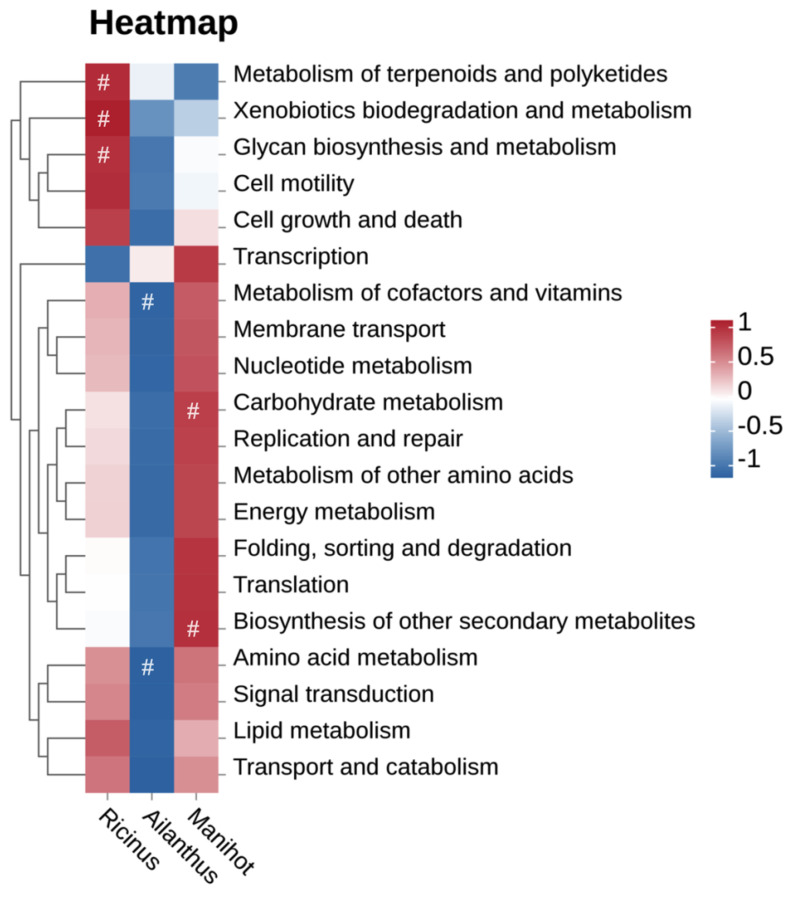
Predicted functional potential of midgut microbiota based on PICRUSt2 analysis. # Indicates KEGG functional pathways with a PICRUSt2-predicted relative abundances greater than 1.

To investigate the potential effects of different food plants on microbial functions, PICRUSt2 analysis was performed on 16S rDNA gene sequencing data. The predicted results showed that most functional prediction categories were associated with metabolic processes (Figure 6). Specifically, the *Ricinus* group exhibited higher predicted relative abundances of gut microbes related to xenobiotics biodegradation and metabolism, metabolism of terpenoids and polyketides, and glycan biosynthesis and metabolism, while the predicted relative abundances of gut microbes associated with carbohydrate metabolism and the biosynthesis of other secondary metabolites were higher in the *Manihot* group. Predicted functional abundances in the *Ailanthus* group were generally lower than those in the other two groups, particularly the metabolism of cofactors and vitamins and lipid metabolism. These results suggest that the diet-induced alterations in midgut microbiota may be associated with nutrient metabolism in *S. ricini*, but this interpretation requires direct functional validation.

### 3.7. Correlation Between Midgut Microbiota, Growth Performance, and Digestive Enzymes

**Figure 7:**
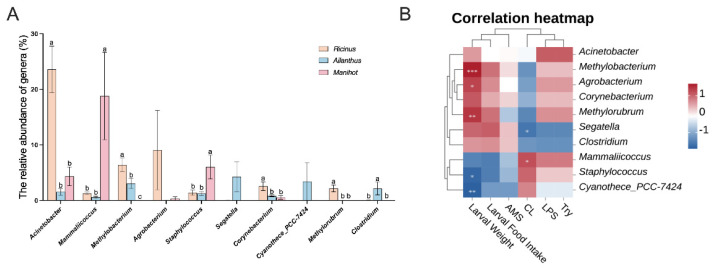
Spearman correlation between midgut microbiota and growth performance indices and digestive enzymes at the genus level. (**A**) Differential bacteria (biomarker) at genus level as determined using LEfSe analysis. Different letters above bars indicate statistically significant differences among the *Ricinus*, *Ailanthus*, and *Manihot* groups; (**B**) Correlation heatmap showing Spearman’s rank correlation coefficients between the relative abundance of differentially abundant genera and host phenotypic traits. Red indicates positive correlations; blue indicates negative correlations. *, *p* < 0.05; **, *p* < 0.01; ***, *p* < 0.001.

Furthermore, microbiota biomarkers at the genus level defined by LEfSe were screened out (Figure 7A) and analyzed by Spearman correlation analysis to investigate relationships between the microbiota and growth performance indices. Regarding growth-related traits, *Methylobacterium*, *Methylorubrum*, and *Agrobacterium* were significantly positively correlated with larval weight, while *Staphylococcus* and *Cyanothece_PCC-7424* were significantly negatively correlated with larval weight. Concerning digestive enzymes, *Mammaliicoccus* showed a significant positive correlation with cellulase activity, whereas *Segatella* was significantly negatively correlated with cellulase activity. We observed a correlation between diet-induced shifts in the midgut microbiota and *S. ricini*’s growth performance.

## 4. Discussion

Diet is a primary determinant of growth, development, and gut microbiota structure in insects. In this study, we observed that three host plant diets, including leaves of *R. communis*, *A. altissima*, and *M. esculenta*, were associated with differences in larval growth performance, digestive enzyme activities, and gut microbiota communities in *S. ricini*. Our findings provide a comprehensive characterization of how diet-driven shifts in the gut microbiota are associated with host digestion and growth performance.

Consistent with previous reports, diet is the main factor affecting the growth performance of *S. ricini* [22,23]. Larvae fed with *R. communis* leaves exhibited better growth performance than those fed with *A. altissima* and *M. esculenta* leaves, as shown by significantly higher body weight on days 2 and 3 and significantly higher weight gain on days 2 and 4. Specifically, the *Ricinus* group showed significantly higher larval weight on day 2 and day 3, food intake on day 2, daily weight gain on day 2, and body weight and weight gain on day 4. Because we did not perform direct phytochemical profiling of the test diets, our interpretations regarding specific leaf compounds remain inferential. Nevertheless, published literature provides a clear baseline for how host-specific nutrients and defensive traits act as selective drivers of the gut environment. *R. communis* leaves are rich in crude protein (39.58–41.70%) and essential minerals [24], offering an optimal substrate for the proliferation of beneficial, nutrient-processing gut microbes. Although *R. communis* contains lipophilic extracts—such as linolenic and linoleic acids which are toxic to non-adapted herbivores [25], and specific phenolic acids [26], these traits likely cultivate a specialized, stable gut microbiota. This adapted microbial community helps *S. ricini* maintain metabolic homeostasis, resulting in increased larval weight and food intake.

Conversely, secondary metabolites in non-host plants can disrupt the gut ecosystem and impair host performance. Although *M. esculenta* leaves have considerable crude potential content (approximately 210 g/kg DM) [27], their anti-nutritional factors, such as tannins and cyanogenic glycosides, correlate negatively with *S. ricini* survival and economic traits [28,29]. Dietary cyanide induces systemic stress and leaves detectable tissue residues in the silkworm [29]. Furthermore, cyanide is known to exert antimicrobial effects that perturb the gut microbiota, causing dysbiosis or shifting microbial functions from digestion toward stress response [30]. Similarly, the potent quassinoids (e.g., ailanthone, chaparrinone, and glaucarubinone) in *A. altissima* act as strong antifeedants and growth inhibitors [31,32]. These compounds can suppress sensitive microbial taxa in the midgut, altering community structure and disrupting the metabolic support the microbiota normally provides for host growth and enzyme activation. We hypothesize that host plant chemical traits (nutrients versus defense compounds) serve as an upstream filter shaping the *S. ricini* gut microbiota. This altered microbiota, in turn, impacts host nutrient absorption and physiological enzyme activities, ultimately leading to the observed variations in larval growth. Future studies should integrate phytochemical analyses (e.g., crude protein, total lipids, tannins, cyanide content, quassinoids) to establish direct links between dietary components, microbiota shifts, and host phenotypes.

Digestive enzymes are crucial for nutrient hydrolysis and insect development [33,34]. Although the activities of α-amylase, cellulase, and trypsin showed no significant differences among the three groups, lipase activity was significantly higher in the *Ricinus* group than in the *Ailanthus* group, while the *Manihot* group showed intermediate levels that did not differ significantly from either group. Lipase plays a vital role in lipid digestion, storage, and mobilization [35], and its activity largely depends on gut pH, diet composition, and dietary requirements [34,36,37]. The elevated lipase activity in the *Ricinus* group compared to the *Ailanthus* group is likely a direct adaptation to the higher fat content in *R. communis* leaves [38], facilitating efficient lipid digestion and contributing to the growth and development of *S. ricini*. The absence of significant changes in α-amylase, cellulase, and trypsin activities across the three groups, together with the differential activity between the *Ricinus* and *Ailanthus* groups, suggests that the dietary switch primarily affected lipid metabolism in larvae fed *R. communis* leaves, while the capacities for starch, cellulose, and protein digestion remained relatively stable under the tested conditions. Our results indicate that food plant type differentially affects the midgut microbial community composition of *S. ricini.* Gut microbiota play important roles in insect growth, nutrient digestion and absorption, and detoxification. Consistent with this, we found that diet is associated with substantial changes in the community structure and diversity of gut microbiota. The abundance and diversity of the gut bacterial community were higher in larvae fed *R*. *communis* and *A. altissima* leaves than in those fed *M. esculenta* leaves. At the phylum level, Bacteroidota, Bacillota, Pseudomonadota, and Cyanobacteriota were dominant. Among them, Pseudomonadota was the most abundant phylum in the *Ricinus* group. This phylum is often associated with beneficial functions in insects, such as carbohydrate degradation and the synthesis of vitamin B or essential amino acids [39,40], potentially correlating with the better growth performance of *S. ricini* fed *R. communis* leaves. In contrast, Cyanobacteriota was the most dominant phylum in the *Manihot* group. These Cyanobacteriota are recognized for their ability to fix atmospheric nitrogen, converting N_2_ into bioavailable ammonium [41]. Although *M. esculenta* leaves contain considerable crude protein (~210 g/kg DM) [27], their high levels of cyanogenic glycosides may limit nitrogen utilization. Therefore, long-term feeding on this plant may impose nitrogen limitations on *S. ricini*. We speculate that the enrichment of diazotrophic Cyanobacteriota in the gut microbiota might provide an additional nitrogen source for the host, potentially as an adaptive response to nutritional constraints.

At the genus level, *Methylobacterium* and *Acinetobacter* were increased in the *Ricinus* group. *Methylobacterium* is involved in the utilization of organic carbon sources and amino acids, and it can degrade plant-derived phenolic and terpenoid compounds, associated with intestinal absorption [42,43,44]. This aligns with the enrichment of the heterotrophic biodegradation and metabolism pathway in this group. *Acinetobacter* has been linked to efficient nutrient absorption in insect guts [45,46], which is correlated with larval growth by improving nutrient utilization. However, *Mammaliicoccus* was significantly increased in the *Manihot* group. The presence of proteinase activity in *Mammaliicoccus* may be associated with aiding the digestion of protein components in the food plants [47]. Silkworms rely on enzymes derived from gut microflora for their nutrition, growth, and development.

Spearman correlation analysis further revealed associations between gut microbial genera and host growth performance as well as digestive enzyme activities in *S. ricini*. In this study, *Methylobacterium*, *Methylorubrum*, and *Agrobacterium*, which were enriched in the *Ricinus* group, showed significant positive correlations with larval weight. Similarly, a study on *Rhoptroceros cyatheae* reported that *Methylobacterium and Methylorubrum* were dominant genera in the larval gut when feeding on different host plants, and their relative abundance varied significantly with host plant species [48]. In contrast, *Staphylococcus* and *Cyanothece_PCC-7424*, which were enriched in the *Manihot* group, showed significant negative correlations with larval weight. Notably, a study on *Spodoptera frugiperda* reported that high concentrations of *Staphylococcus sciuri* significantly decreased larval survival rate and body weight [49], which is consistent with the negative correlation observed in the present study. Regarding digestive enzyme activities, *Mammaliicoccus* showed a significant positive correlation with cellulase activity, suggesting that this genus may possess cellulolytic potential. *Segatella* showed a significant negative correlation with cellulase activity; this genus was enriched in the *Ailanthus* group, and this negative correlation may be associated with the lower growth performance observed in this group, although the underlying mechanism remains unclear. In summary, genera enriched in the *Ricinus* group (e.g., *Methylobacterium*, *Methylorubrum*, *Agrobacterium*) were positively associated with growth performance, whereas *Staphylococcus* enriched in the *Manihot* group was negatively associated with larval weight, and the positive correlation between *Mammaliicoccus* and cellulase activity suggests that this genus may be involved in cellulose decomposition. These correlation patterns suggest that diet-induced shifts in gut microbiota are associated with growth performance and digestive enzyme activities in *S. ricini*. However, these findings are correlational, and causal relationships require functional validation using gnotobiotic insects, antibiotic treatments, or microbiota transplantation.

## 5. Limitations

We acknowledge several limitations in this study. We did not chemically analyze the host plant leaves. We interpret differences in larval growth, enzyme activities, and gut microbiota across diet groups, but without actual measurements of nutrients or defensive compounds in the three plants, those interpretations are largely speculative. We do cite published work to support the known differences among *R. communis*, *A. altissima*, and *M. esculenta*, but direct chemical profiling of the leaves we used would be needed to strengthen our conclusions.

Another issue is the lack of baseline gut microbiota data before the diet switch. All larvae were fed *R. communis* from the 1st to the 4th instar and then randomly assigned to the three diets at the 5th instar. Randomization should distribute any initial individual differences evenly across groups. However, the microbiota shaped by *R. communis* during early instars may have persisted after the switch, and this carryover effect would apply to all groups equally. Still, our β-diversity analysis clearly separated the three groups, suggesting that short-term diet shifts did change the gut microbiota. That said, without day-0 baseline samples, we cannot fully tell whether the observed differences came from the new diets or from the lingering effect of the old one.

We also recognize a limitation regarding functional inference. We used 16S rDNA sequencing and PICRUSt2, which only provide indirect predictions of microbial function. This approach cannot tell us whether the predicted metabolic pathways are actually active in the gut. Future work using metatranscriptomics or metaproteomics would be needed to directly measure function, and gnotobiotic models or microbiota transplantation would be required to test causality.

Despite these limitations, this study offers a detailed picture of how diet shifts alter the gut microbiota of *S. ricini* and provides testable hypotheses for future mechanistic studies.

## 6. Summary

In summary, *Samia ricini* larvae fed on *R. communis* leaves showed better growth performance. Midgut lipase activity was significantly higher in the *Ricinus* group than in the *Ailanthus* group, with the *Manihot* group showing intermediate activity that did not differ significantly from either. Different host plants were associated with distinct gut microbial community structures, including bacterial richness and diversity. Functional predictions suggested that diet-associated shifts in microbiota may relate to specific metabolic pathways, such as terpenoid/polyketide metabolism in the *Ricinus* group and carbohydrate metabolism in the *Manihot* group. Taken together, these findings indicate a potential link between diet, gut microbiota composition, and host growth performance, further supporting the important role diet plays in shaping insect gut microbial communities.

## Figures and Tables

**Table 1:** Experimental design.

Group	Instar	Diet	Number of Larvae	Number of Replicates	Sampling Points
entry 1	1st–4th	*R. communis*	-	-	-
*Ricinus*	5th	*R. communis*	30	3	Day 3
*Ailanthus*	5th	*A. altissima*	30	3	Day 3
*Manihot*	5th	*M. esculenta*	30	3	Day 3

## Data Availability

The raw data supporting the conclusions of this article will be made available by the authors on request.
